# Supplementary material for: Changes in gene expression of cervical collagens, metalloproteinases, and tissue inhibitors of metalloproteinases after partial cervical excision-induced preterm labor in mice
Source: PLoS One. 2021 Apr 14;16(4):e0250108. doi: 10.1371/journal.pone.0250108 (PMC8046204; doi:10.1371/journal.pone.0250108)
Supplement: Attachment — Submitted filename: Response to Reviewers.docx [file pone.0250108.s002.docx]

Journal Requirements:
 
 
When submitting your revision, we need you to address these additional requirements.
 
1. Please ensure that your manuscript meets PLOS ONE's style requirements, including those for file naming. The PLOS ONE style templates can be found at
<https://journals.plos.org/plosone/s/file?id=wjVg/PLOSOne_formatting_sample_main_body.pdf> and
<https://journals.plos.org/plosone/s/file?id=ba62/PLOSOne_formatting_sample_title_authors_affiliations.pdf>

🡪 We revised a manuscript according to PLOS ONE’s style requirements.

2. We note that you have indicated that data from this study are available upon request. PLOS only allows data to be available upon request if there are legal or ethical restrictions on sharing data publicly. For more information on unacceptable data access restrictions, please see <http://journals.plos.org/plosone/s/data-availability#loc-unacceptable-data-access-restrictions>.
In your revised cover letter, please address the following prompts:
a) If there are ethical or legal restrictions on sharing a de-identified data set, please explain them in detail (e.g., data contain potentially sensitive information, data are owned by a third-party organization, etc.) and who has imposed them (e.g., an ethics committee). Please also provide contact information for a data access committee, ethics committee, or other institutional body to which data requests may be sent.
b) If there are no restrictions, please upload the minimal anonymized data set necessary to replicate your study findings as either Supporting Information files or to a stable, public repository and provide us with the relevant URLs, DOIs, or accession numbers. For a list of acceptable repositories, please see <http://journals.plos.org/plosone/s/data-availability#loc-recommended-repositories>.
We will update your Data Availability statement on your behalf to reflect the information you provide.

🡪 The comment on the data set has been inserted in coverletter.
3. Thank you for stating the following in the Funding Section of your manuscript:
"This research was supported by the Basic Science Research Program through the
39 National Research Foundation of Korea, which is funded by the Ministry of Science and ICT40 (2014R1A1A1002300 and 2018R1D1A1B07)."
We note that you have provided funding information that is not currently declared in your Funding Statement. However, funding information should not appear in the Acknowledgments section or other areas of your manuscript. We will only publish funding information present in the Funding Statement section of the online submission form.
Please remove any funding-related text from the manuscript and let us know how you would like to update your Funding Statement. Currently, your Funding Statement reads as follows:
"The funders had no role in study design, data collection and analysis, decision to publish, or preparation of the manuscript."

Please include your amended statements within your cover letter; we will change the online submission form on your behalf.

🡪 We removed funding statement from the manuscript. Funding information will be presented in the financial disclosure section of the submission system as follows. "This research was supported by the Basic Science Research Program through the National Research Foundation of Korea, which is funded by the Ministry of Science and ICT40 (2014R1A1A1002300 and 2018R1D1A1B07). The funders had no role in study design, data collection and analysis, decision to publish, or preparation of the manuscript."

[Note: HTML markup is below. Please do not edit.]

Reviewers' comments:

Reviewer's Responses to Questions
**Comments to the Author**

1. Is the manuscript technically sound, and do the data support the conclusions?

The manuscript must describe a technically sound piece of scientific research with data that supports the conclusions. Experiments must have been conducted rigorously, with appropriate controls, replication, and sample sizes. The conclusions must be drawn appropriately based on the data presented.
Reviewer #1: Partly
Reviewer #2: Yes
 
2. Has the statistical analysis been performed appropriately and rigorously?
 
Reviewer #1: Yes
Reviewer #2: Yes
 
3. Have the authors made all data underlying the findings in their manuscript fully available?

The [PLOS Data policy](http://www.plosone.org/static/policies.action#sharing) requires authors to make all data underlying the findings described in their manuscript fully available without restriction, with rare exception (please refer to the Data Availability Statement in the manuscript PDF file). The data should be provided as part of the manuscript or its supporting information, or deposited to a public repository. For example, in addition to summary statistics, the data points behind means, medians and variance measures should be available. If there are restrictions on publicly sharing data—e.g. participant privacy or use of data from a third party—those must be specified.
 
Reviewer #1: Yes
Reviewer #2: Yes
 
4. Is the manuscript presented in an intelligible fashion and written in standard English?

PLOS ONE does not copyedit accepted manuscripts, so the language in submitted articles must be clear, correct, and unambiguous. Any typographical or grammatical errors should be corrected at revision, so please note any specific errors here.
 
Reviewer #1: Yes
Reviewer #2: Yes
 
5. Review Comments to the Author

Please use the space provided to explain your answers to the questions above. You may also include additional comments for the author, including concerns about dual publication, research ethics, or publication ethics. (Please upload your review as an attachment if it exceeds 20,000 characters)
 
Response to Reviewer

Reviewer #1: To test the changes in the levels of cervical collagens MMPs and TIMPs, it would be convenient for the authors to perform immunoblot with the available samples.

- We truly aware of your critical advice. Immunoblot would make our data more understandable however we tried to quantify gene expression of cervical collagens, MMPs and TIMPs using our cervical excision mouse model. Now our team don’t have available samples because this project already ended. Spite of the absence of protein expression data, this study might be meaningful as a pilot study. This limitation has been inserted as a limitation in Discussion section. Thank you for your understanding.

In the line 121, when the authors mention their previous studies in the methods, they should include a reference if they are published.

- We added the reference of our prior experiment.
- 11. Ahn KH, Jeong HC, Kim HY, Kang D, Hong SC, Cho GJ et al. Relationship between partial uterine cervical tissue excision and preterm birth: an experimental animal study. Am J Perinatol. 2017;34:1072–1077.

When the authors explain the cervical excision, it would be convenient included if any kind of analgesia has been administered to the animals after the surgery.

- For the cervical excision surgery, xylazine (Rompun®, Bayer, Toronto, Canada) is administered intravenously immediately before surgery. This information has been added in Method section. (Line 102-103)

In the results, in line 175 authors should include the SD in the gestational periods.

- As per comment, we have added the SD value in the gestational periods.

On the y-axis of the figures, authors should include the name of the collagenase, MMP or TIMs studied instead of fold.

- We included the name of the collagenase, MMP or TIMPs on the y-axis of the figures as suggested instead of fold.

In figure 2 authors should check the letters off the graphs

- We corrected as suggested in Figure 2.

Reviewer #2: In the present manuscript the authors show the changes in the expression of cervical collagens, metalloproteinases and tissue inhibitors of metalloproteinases that are produced in a model of preterm labor induced by a partial cervical excision in mice. Thus, the authors justify that these components and proteins participate in the remodeling after a spontaneous premature birth after inflation added to a cervical excision.

However, the study lacks of novelty. The comments are shown below.

Introduction

Minor comments

- The authors should show that it is not only TIMPs that are regulating MMPS activity. There are other proteins that can participate in this signaling pathway, such as CD147, also known as EMMPRIN (Extracellular Matrix Metalloproteinase INducer). Line 94.

- As per comment, we added EMMPRIN as a regulator of MMPs with a reference.
- 8. Li K, Nowak RA. The role of basigin in reproduction. Reproduction. 2019 Sep 1:REP-19-0268.R1.

- Paragraph staring from line 86 to 90 is necessary to remove it. Experimentation and research on humans are totally forbidden and does not need to be discussed.\

- As per comment, we deleted sentences in line 86 to 90.

Methods

- What is the concentration of LPS given to the mice?

- We gave 100 μg of LPS in each mice. We added more detailed explanation for volume of LPS concentration in Material and Methods section.

- Why was the administration of LPS done on the 16th day gestational?

- Preterm birth is defined before gestational day 18 in mouse therefore LPS induction is usually performed on gestational day 15-17 to induce preterm birth. In addition, this protocol using LPS induced preterm birth is usually performed on gestational day 15-17 in previous studies. Following reference shows similar protocol.
- The Local and Systemic Immune Response to Intrauterine LPS in the Prepartum Mouse. Edey LF, O'Dea KP, Herbert BR, Hua R, Waddington SN, MacIntyre DA, Bennett PR, Takata M, Johnson MR. Biol Reprod. 2016 Dec;95(6):125. doi: 10.1095/biolreprod.116.143289. Epub 2016 Oct 19.

Results

- Figure 2 shows graphically significant differences in the expression levels of MMP2 and TIMP-1 that the authors do not comment.

- We made comments on MMP2 and TIMP-1 in results line 167-173 as follows.
- There were no significant differences in the expression of MMP-2 and TIMP-1 among the experimental groups, but MMP-2 expression showed an increasing trend in the partial cervical tissue excision, administration of LPS, and partial cervical tissue excision plus administration of LPS groups compared to the sham group. In addition, TIMP-1 expression did not show significant differences although its expression in the partial cervical tissue excision plus administration of LPS group was lower compared to the other groups.

- Why does the LPS group have a low n compared to the other groups?

- Thank you for your comment. To minimize the conditions affecting the experiment, mouse experiments were conducted at once. The mice were randomly assigned to one of four groups and distributed 10 mice per group. Mice was bred in the eighth week, and mice that were not pregnant for more than eight weeks were sacrificed. The LPS groups had fewer mice pregnant at week 8 compared to other groups.

- Figure 2, the graphics are incorrectly listed

- We corrected Figure 2.

- Due to the fact that there are a wide number of Metalloproteinases and TIMPs involved in remodeling processes in response to an injury, the authors should screen for these proteins (MMP-9, TIMP-2, MMP-13...)

- We appreciate suggestion on screening for MMP-9 and other MMPs and TIMPs which have been widely studied in abortion, preeclampsia, and preterm birth. We conducted this study to elucidate the function of MMP-2, MMP-7, MMP-14 and TIMP-1 which are closely related to Col4 and Col5 utilizing our cervical excision model. We focused on the Col4 and Col5 and related enzymes in this study.

- To complete the study, the authors must show the protein expression of the MMPs through Western Blot and must also show the activity of these proteins using a Zymography

- Thank you for your comment. Although the current study is focusing on the gene quantification of collagens, MMPs and TIMPs of partial cervical excision plus LPS, authors agree with the reviewer’s viewpoint. We addressed your points as a limitation in the Discussion line 284-285.

- In relation to line 299 to 301, the authors should use for basic techniques like Hematoxylin/Eosin or Masson's Trichrome stain to show a change in the cervix ECM before/after excision

- Thank you for your comment. In our study, the cervical excision and LPS injection is one treatment option, which is compared to sham group. Therefore, change in the collagen and its enzymes after the treatment (cervical excision and LPS injection as a whole) is important. We have a future plan for the study on ECM change before vs. periodic follow-up after excision. Your advice is commented as a limitation in the main text.

6. PLOS authors have the option to publish the peer review history of their article ([what does this mean?](https://journals.plos.org/plosone/s/editorial-and-peer-review-process#loc-peer-review-history)). If published, this will include your full peer review and any attached files.


If you choose “no”, your identity will remain anonymous but your review may still be made public.

**Do you want your identity to be public for this peer review?** For information about this choice, including consent withdrawal, please see our [Privacy Policy](https://www.plos.org/privacy-policy).
 
Reviewer #1: No
Reviewer #2: No
 

[NOTE: If reviewer comments were submitted as an attachment file, they will be attached to this email and accessible via the submission site. Please log into your account, locate the manuscript record, and check for the action link "View Attachments". If this link does not appear, there are no attachment files.]

While revising your submission, please upload your figure files to the Preflight Analysis and Conversion Engine (PACE) digital diagnostic tool, <https://pacev2.apexcovantage.com/>. PACE helps ensure that figures meet PLOS requirements. To use PACE, you must first register as a user. Registration is free. Then, login and navigate to the UPLOAD tab, where you will find detailed instructions on how to use the tool. If you encounter any issues or have any questions when using PACE, please email PLOS at [figures@plos.org](mailto:figures@plos.org). Please note that Supporting Information files do not need this step.
 
 


In compliance with data protection regulations, you may request that we remove your personal registration details at any time. [(Remove my information/details)](https://www.editorialmanager.com/pone/login.asp?a=r). Please contact the publication office if you have any questions.
