# Supplementary material for: Changes in gene expression of cervical collagens, metalloproteinases, and tissue inhibitors of metalloproteinases after partial cervical excision-induced preterm labor in mice
Source: PLoS One. 2021 Apr 14;16(4):e0250108. doi: 10.1371/journal.pone.0250108 (PMC8046204; doi:10.1371/journal.pone.0250108)
Supplement: Attachment — Submitted filename: Response to editor and reviewers.docx [file pone.0250108.s003.docx]

Response to editor

- **Thank you for your positive decision.**

Response to reviewers

Reviewer #1: Authors should check in line 117 of the new manuscript if the reference to the previous study is number 10 or 11 "The method of animal experimentation is described in our previous study".

- **As per your comment, we have corrected the number of the reference. (10 🡪 11)**

Reviewer #2: All comments have been addressed correctly. The authors have made the manuscript better and improved the quality of the work.

- **Thank you for your positive answer.**
